# Adapting an Ant Colony Metaphor for Multi-Robot Chemical Plume Tracing

**DOI:** 10.3390/s120404737

**Published:** 2012-04-12

**Authors:** Qing-Hao Meng, Wei-Xing Yang, Yang Wang, Fei Li, Ming Zeng

**Affiliations:** Institute of Robotics and Autonomous Systems, School of Electrical Engineering and Automation, Tianjin University, No. 92, Weijin Road, Tianjin 300072, China; E-Mails: weixing@tju.edu.cn (W.-X.Y.); raynor63@163.com (Y.W.); lifei@tju.edu.cn (F.L.); zengming@tju.edu.cn (M.Z.)

**Keywords:** ant colony metaphor, chemical plume tracing, time-varying airflow environment, multiple robots, spiral surge

## Abstract

We consider chemical plume tracing (CPT) in time-varying airflow environments using multiple mobile robots. The purpose of CPT is to approach a gas source with a previously unknown location in a given area. Therefore, the CPT could be considered as a dynamic optimization problem in continuous domains. The traditional ant colony optimization (ACO) algorithm has been successfully used for combinatorial optimization problems in discrete domains. To adapt the ant colony metaphor to the multi-robot CPT problem, the two-dimension continuous search area is discretized into grids and the virtual pheromone is updated according to both the gas concentration and wind information. To prevent the adapted ACO algorithm from being prematurely trapped in a local optimum, the upwind surge behavior is adopted by the robots with relatively higher gas concentration in order to explore more areas. The spiral surge (SS) algorithm is also examined for comparison. Experimental results using multiple real robots in two indoor natural ventilated airflow environments show that the proposed CPT method performs better than the SS algorithm. The simulation results for large-scale advection-diffusion plume environments show that the proposed method could also work in outdoor meandering plume environments.

## 1. Introduction

Olfaction is widely used by many animals for searching for food, finding mates, exchanging information, and evading predators. Such animals could be trained to help humans seek appointed gas sources. For example, it is well known that specially trained dogs are often used to find bombs, mines, drugs, and even people buried by avalanches [1]. However, it takes a long time to train such animals. In addition, animals are prone to fatigue so they cannot efficiently work for long periods. Moreover, animals are not suitable for working in dangerous areas (e.g., where there are toxic gases).

Inspired by the odor source localization (OSL) abilities of many animals, in the early 1990s researchers started to build mobile robots with such abilities to replace trained animals [2–4]. Compared to animals, robots could be deployed quickly and maintained at low cost. In addition, robots could work for long periods without fatigue, and most importantly, they can enter dangerous areas. It is expected that mobile robot based OSL will play increasing roles in areas such as judging toxic or harmful gas leakage location, checking for contraband (e.g., heroin), searching for survivors in collapsed buildings, humanitarian de-mining, and fighting against terrorist attacks.

The behavior based OSL task can be decomposed into three sub-procedures, namely plume finding, plume traversal, and source declaration, according to Hayes *et al.* [5], or four sub-procedures according to Li *et al.* [6], namely finding a plume, tracing the plume, reacquiring the plume, and declaring the source. During the initial phase, contact is made with a plume. Once the plume is detected the robot traces the chemical toward its source. In the final phase the robot locates the source. To our knowledge, until now most research related to OSL focuses on plume tracing, which may be why mobile robot based OSL is also called chemical plume tracing (CPT) [7,8]. The way in which a robot performs each of these phases depends upon the nature of the chemical plume, and the resources available to the robot.

The commonly used methods for finding the chemical plume consist of zigzag [4,6] and spiral [5] motions. Experimental comparison of the spiral, up-flow and down-flow zigzag strategies conducted in outdoor natural airflow environments shows that all of these strategies present a high success rate, with the down-flow zigzag strategy consuming the shortest time in finding a plume [9].

The traditional plume tracing methods include chemotaxis [10] and anemotaxis [4], which are biologically inspired algorithms. The custom algorithms such as fluxotaxis [8] and infotaxis [11] have also been proposed and tested. Several insect-inspired chemical plume-tracing algorithms, including surge anemotaxis, bounded search and counterturning have been compared using a mobile robot [12]. Four reactive robot chemotaxis algorithms, observed in the bacterium *E. coli*, the silkworm moth *Bombyx mori*, and the dung beetle *Geotrupes stercorarius* as well as a gradient-based algorithm, have also been implemented and evaluated [10]. Li *et al.* [13] presented a particle filter algorithm for odor source localization in outdoor time-variant airflow environments.

To identify the gas source, Lilienthal and his colleagues [14] adopted an artificial neural network and support vector machine to classify whether or not an object was a gas source from a series of concentration measurements recorded while the robot performed a rotation maneuver in front of it. Li and his colleagues [6] designed a source declaration logic based on analysis, Monte Carlo simulation, and results of initial field experiments. Li and Meng [15] put forward a three-step single odor source declaration method. Experimental results in indoor airflow environments using three small mobile robots validated the feasibility.

Compared with the single-robot search, multiple robots might have at least two advantages: the expected search time could be decreased; and multi-robot systems could provide a greater robustness against hardware failures. The particle swarm optimization (PSO) algorithm was tested using computer simulation by Jatmiko [16] and Marques [17] using the plume models developed by Farrell [18] and Nielsen [19], respectively. Byrne and his colleagues [20] described a system of miniature mobile robots and the algorithms used to demonstrate cooperative plume tracing and source localization. The algorithm was implemented on a group of miniature mobile robots capable of measuring temperature plumes. Meng *et al.* [21] proposed a probability PSO (P-PSO) algorithm for multi-robot based OSL. The P-PSO algorithm used probability to express fitness function. The results of real-robot experiments in ventilated indoor environments and simulations for large-scale advection–diffusion plume environments demonstrated the feasibility and advantage of the proposed P-PSO algorithm. Spears and her colleagues [22] proposed a multi-robot CPT algorithm called fluxotaxis that follows the gradient of the chemical mass flux to locate a chemical source emitter. Ferri and his colleagues [23] used a biologically-inspired algorithm called Searching Pollutant Iterative Rounding Algorithm (SPIRAL) with a Multi-robot for Odor Monitoring (MOMO) platform to localize a gas source in an indoor environment with no strong airflow. Meng *et al.* [24] applied an improved ant colony optimization (ACO) algorithm to multi-robot odor-plume tracing in indoor airflow environments, and real robot experiments demonstrated its feasibility.

Multi-robot based OSL has not been well studied and has mostly been restricted to simulated robots and simulation environments. To our knowledge, only a few publications have discussed the CPT problem with multiple real robots. Hayes [5] proposed a spiral surge (SS) strategy for multiple robots CPT with real robot hardware. Several fans were used to produce an artificial wind field. Lytridis and his colleagues [25] combined the biologically inspired chemotaxis strategy with biased random walking (BRW) strategy to form a chemo-BRW algorithm for multi-robot plume tracing with three BIRAW robots. A Gaussian-shaped odor field was created using a fan. Lochmatter *et al.* [26] introduced a crosswind formation algorithm for chemical plume tracking and carried out experiments in a wind tunnel with laminar airflow. Marjovi and Marques [27] presented a cooperative distributed approach for searching odor sources in unknown structured environments with multiple mobile robots.

The gas plume in real airflow environments is patchy and/or meandering due to the turbulence-dominated gas molecules dispersion. In addition, local concentration maxima caused by large eddies often exist in indoor environments, especially in corners. Therefore, tracing such a dynamic gas plume down to its source is not a trivial task.

The main motivation of our research was to adapt ant colony metaphor for the multi-robot CPT problem in time-varying airflow environments. It is well known that, owing to its inherent features such as a highly efficient form of best-path exploitation (pheromone detection) and a sensible mechanism for exploration (probabilistic path selection), the traditional ACO algorithm proposed by Dorigo [28] has been successfully applied to combinatorial optimization problems in discrete domains. Since the search area is physically continuous and the gas plume is time-variant (patchy and/or meandering), the CPT is actually a dynamic optimization problem in continuous domains. Therefore, the traditional ACO algorithm cannot be directly copied to the multi-robot CPT problem.

To adapt the ant colony metaphor to the multi-robot CPT problem, the two-dimensional continuous search space is discretized into grids, and the virtual pheromones are released in the grids by the robots. The virtual pheromone is updated using both the gas concentration and wind information. To prevent the adapted ACO algorithm from being trapped prematurely in a local optimum, the upwind surge behavior is adopted by the robots with relatively higher gas concentration in order to explore in more areas.

The adapted ACO combined with upwind surge (AACO+US for short) is executed iteratively. At every iteration step, the robots are dynamically divided into two subgroups according to the sampled concentrations. The subgroup with relatively lower concentrations is coordinated by the adapted ACO algorithm to move toward the high-pheromone areas while guaranteeing that distances between robots remain small, meanwhile the other subgroup with relatively higher concentrations searches upwind to explore the plume in more area and thus prevents them from being trapped in local optima.

The proposed AACO+US and the comparative SS algorithms have been verified in two different indoor time-varying airflow environments using real-robot hardware platforms. The CPT performances (including the searching efficiency and success rate) of the two algorithms are compared. The effect of robots' number on the two CPT algorithms' performances is also presented. The possible reasons leading to unsuccessful CPTs are also discussed. The proposed CPT algorithm is also conducted in simulated large-scale advection–diffusion plume environments in order to figure out whether it could cope with the plume meander problem.

The remainder of this paper is organized as follows: in Section 2, the continuous space representation for multi-robot CPT is described. The proposed AACO+US algorithm framework is outlined and explained in Section 3. The comparative algorithm, *i.e.*, the SS algorithm, is briefly introduced in Section 4. Section 5 presents the infrastructures and the CPT performance evaluation indexes for the real-robot experiments. The real-robot experimental results are given and discussed in Section 6. Section 7 shows the CPT simulation, followed by the conclusions presented in Section 8.

## 2. Continuous Space Representation

The discrete versions of the ACO algorithm are able to handle highly constrained order-based problems, and there are well-defined paths for transitions between nodes in discrete domains. For the CPT problem, the given searching area is physically continuous, owing to the fact that there are no well-defined “nodes” or “edges”, so the traditional ACO cannot be directly used for multi-robot CPT problem unless some kind of order-based representation is invented.

**Figure 1.**
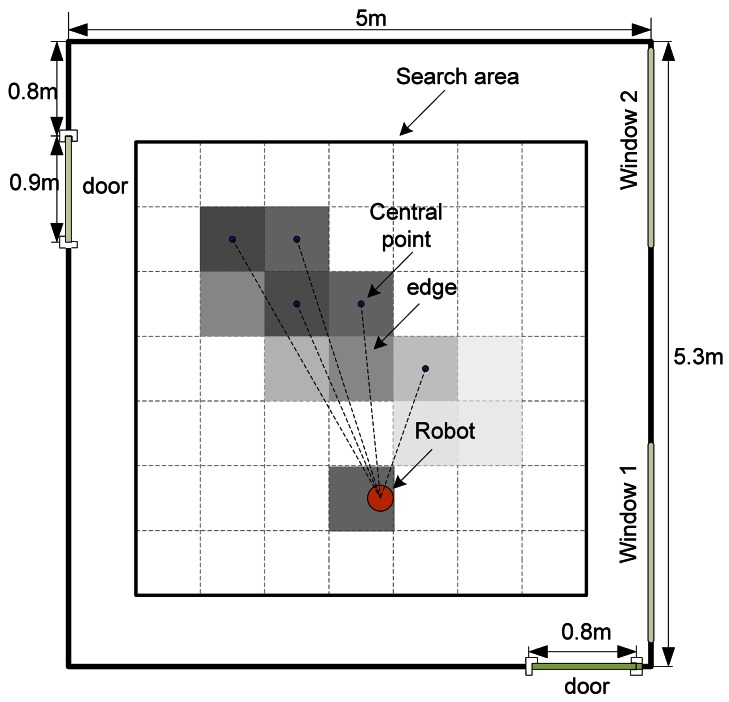
Discretization of the CPT searching environment. The search area is divided into a number of grids. The gray-scale of each grid represents the pheromone value deposited into that grid. Darker color indicates higher pheromone value. The straight dashed lines connecting the robot and the central points are interpreted as edges in the traditional ACO. The robot performing the adapted ACO strategy chooses one of the central points as its temporary goal and moves toward it.

To adapt the ant colony metaphor to the multi-robot CPT problem, the two-dimension continuous search area is divided into numerous square grids (see Figure 1). The grid size is set to 20 cm × 20 cm and 50 cm × 50 cm in indoor and outdoor environments, respectively. The two different grid sizes are set considering the real dimensions of our robots designed for indoor and outdoor environments. The geometric central point of each grid, corresponding to the node in the traditional ACO, has a virtual pheromone value. Every central point is perceived as a feasible solution of the CPT problem, *i.e.*, the possible gas source location. The straight dashed lines connecting the robot to the central points form the edges in the adapted ACO. It should be noted that the pheromone in the adapted ACO is deposited in the “nodes” instead of “edges”. For the convenience of description, we also say that the pheromone is deposited in the grid.

For the multi-robot CPT problem, both the sampled gas concentration and wind information are considered in the pheromone update (details can be seen in Section 3). The global pheromone distribution map is shared by all the robots via communication in the adapted ACO, while in the traditional ACO only pheromones deposited on the edges connected to the current node are known. Because the search area in the CPT problem is continuous, any grid is directly accessible to all robots regardless of the physical distance. Thus, all the grids are connected to current robot in a sense. Each robot is assigned a taboo list, in which the maximal-pheromone grids the robot has passed through are recorded.

## 3. AACO+US Algorithm

**Figure 2.**
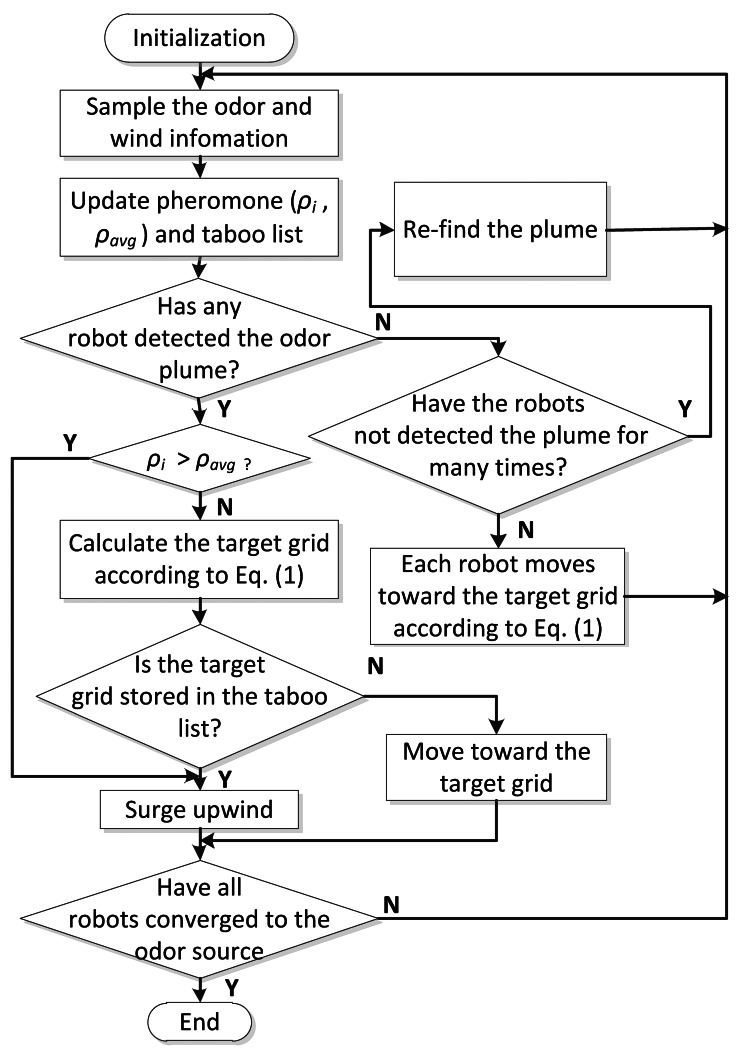
The flowchart of the proposed AACO+US algorithm. The variables *ρ_i_* and *ρ_avg_* stand for the virtual pheromone in the *i*th grid and the average pheromone, respectively.

Figure 2 shows the flowchart of the proposed AACO+US algorithm. At the initial phase, the robots move toward different directions to find plume. The robots measure the wind and gas information, and the pheromones that reflect the newly sampled concentrations are calculated and deposited in the corresponding grids. The robots “deposit” pheromone by modifying appropriate pheromone variables. At the decision-making phase (see the Section 3.1), the robots are dynamically divided into two subgroups according to the pheromones of the grids in which they are located. The goal point is allocated to each robot. When the robots arrive at new positions where the wind and gas information are sampled, the pheromone map and taboo list are updated accordingly. If no pheromone has been detected for several times (five times in our experiments), the robots will scatter to move upwind to re-find the plume.

### 3.1. Decision Making

The multiple robots' motion is coordinated via the AACO+US algorithm. At first, the average pheromone of the grids in which the robots are located is calculated (*ρ_avg_* in Figure 2). Then each robot compares the pheromone of the grid where it is located (*ρ_i_* in Figure 2) with the average value. The robots are divided into two subgroups according to the results of the comparison. The higher-pheromone subgroup (the robots located in the grids with higher pheromone than the average value) surges upwind to explore more areas, expecting to detect even higher concentration and avoid being trapped in local optima prematurely. The lower-pheromone subgroup, by contrast, performs the adapted ACO strategy to move toward the maximal-pheromone areas while guaranteeing that the distance between the robots remains small, in order to keep the historical and colonial optimal solutions. Each robot of this subgroup chooses (via roulette wheel) one of the five grids with the highest pheromone value as its goal. The transfer probability that the robot *k* in the *i*th grid moves toward the *j*th grid at time *t* is determined by Equation (1):

(1-a) $$\begin{array}{cc} p_{ij}^{k}(t)=\frac{{\left[\tau_{ij}(t)\right]}^{\alpha }{\left[\eta_{ij}(t)\right]}^{\beta }}{\underset{s\in \max(t)}{\text{∑}}{\left[\tau_{is}(t)\right]}^{\alpha }{\left[\eta_{is}(t)\right]}^{\beta }}, & j\in \max(t), \end{array}$$

(1-b) $$\tau_{ij}(t)=e^{\rho_{j}(t)/C},$$

(1-c) $$\eta_{ij}(t)=e^{-d_{ij}(t)/D},$$

where:

*τ_ij_*(*t*) and *η_ij_*(*t*) represent the pheromone and heuristic information, respectively,
*ρ_j_*(*t*) is the pheromone of the *j*th grid at the time *t*,
d*_ij_*(*t*) stands for the Euclidian distance between the *i*th and *j*th grids,
*C, D, α* and *β* are constants, and
max(*t*) stores the indices of five maximal-pheromone grids at the current time *t*.

Here *p^k^_ij_*(*t*) has the same expression as that of the traditional ACO algorithm. The effect of the item *τ_ij_*(*t*) in the adapted ACO is to make the robots move according to the pheromone, while the item *η_ij_*(*t*) makes the robots move according to the distance information.

### 3.2. Pheromone Deposit and Update

The given search area is divided into *M* × *N* grids, and the detailed values of *M* and *N* depend on the dimensions of environments and robots. At the very beginning, the pheromone in each grid is set to zero. The pheromone deposited in the *q*th grid (*q* ∈ *pos*(*t*), *pos*(*t*) stores the indices of grids in which the robots are located) is calculated as follows:

(2) $$\rho_{q}(t)=\frac{c_{\text{avg}}(t)}{c_{\max}},$$

where *c_avg_* is the averaged gas concentration detected by the robot in the *q*th grid at the time *t*; and *c*_max_ is the upper limit of the concentration that the gas sensor can measure, thus the value of the pheromone *ρ_q_*(*t*) is between 0 and 1.

The pheromone updates include global update and local update. The global update mainly considers gas concentration (*i.e.*, the virtual pheromone) evaporation in all the grids caused by the wind speed, which can be expressed as follows:

(3) $$\left\{ \begin{array}{l} \rho_{i}(t+\Delta t)=[1-\delta (t)]\rho_{i}(t)\\ \delta (t)=\left|\frac{V_{\text{avg}}(t)}{V_{\max}(t)}\right| \end{array}\right.i=1,2,\ldots ,M\times N,$$

where *ρ_i_*(*t*) stands for the pheromone map at the time *t; ρ_i_*(*t* + Δ*t*) indicates the updated pheromone map at the time *t* + Δ*t*, Δ*t* denotes the time step; *V_avg_*(*t*) represents the average wind strength measured by the robots at the time *t; V*_max_(*t*) is the maximal wind strength up to the current time *t* measured by the robots. Hence the value of *δ*(*t*) is smaller than 1.

The local pheromone update only considers the grids in which the robots are located, and it uses both the historical and new pheromones. The newly deposited pheromone weighs more heavily in the local update, which can be expressed as follows:

(4-a) $$\rho_{q}(t+\Delta t)\leftarrow \omega (t)\rho_{q}(t)+(1-\omega (t))\rho_{q}(t+\Delta t),\;q\in \text{pos}(t),$$

(4-b) $$\omega (t)=0.6+\frac{\sigma_{f}(t)}{\sigma_{f\_\max}(t)},$$

where *ρ_q_*(*t* + Δ*t*) in the LHS of Equation (4-a) is the updated pheromone in the *q*th grid at the time *t* + Δ*t; ρ_q_*(*t*) stands for the pheromone in the *q*th grid left by the new robot at the time *t*, which is calculated by Equation (2); *ρ_q_*(*t* + Δ*t*) in the RHS of Equation (4-a) is the pheromone globally updated using Equation (3); *σ_f_*(*t*) and *σ_f__*_max_(*t*) in Equation (4-b) stand for the wind-direction variance at the time *t* and the maximal wind-direction variance up to the time *t*, respectively. If the value of *ω*(*t*) is bigger than 1, *ω*(*t*) is set to 1. The newly deposited pheromone, however, weighs more heavily because it is thought that the pheromone left by the new robot is more believable. Here we set a constant 0.6 in *ω*(*t*) to ensure that the weight of the new pheromone is 0.6 at least. Larger *σ_f_*(*t*) indicates more violent fluctuation in wind direction, and the historical pheromone becomes less credible, so less weight is distributed to it.

### 3.3. Taboo List Update

Each robot has a *taboo list* recording the maximal-pheromone grids that the robot has passed through. To make the robot search more areas and avoid being trapped in a local concentration optimum, the robot moves upwind if the maximal-pheromone grid toward which the robot would move is one of the grids stored in the taboo list. In each iterative loop, both the taboo lists of the robots and the maximal-pheromone grids are updated. The grids stored in the taboo list that do not belong to the newest maximal-pheromone grids are removed.

## 4. The Comparative SS Algorithm

The AACO+US strategy proposed in this paper is compared with the SS algorithm proposed by Hayes [5] to verify the performance of the former algorithm. The traditional SS algorithm finds the plume by an initial outward spiral search pattern (SpiralGap1). When the robot detects an odor packet with a concentration value higher than a certain threshold during spiraling, the wind direction is sampled and the robot moves upwind for a certain distance (StepSize). If the robot detects another odor packet during surge, it resets the surge distance but does not resample the wind direction. As the robot has reached the surge distance, it behaves a tighter spiral casting (SpiralGap2) for another plume hit. If the robot re-detects an odor packet, it repeats the surge behavior. If there is no odor packet detected in a set time (CastTime), a plume re-finding behavior (less local spiral) is performed.

The detection threshold, which determines the behavior of the robot, is a key parameter in the SS algorithm. The amplitude threshold for odor detection in [5] was set at 4 times the baseline standard deviation (recorded from 10,000 samples taken at an average rate of 85 Hz). Considering that odor concentration accumulates with time in indoor environments, however, a moving average concentration value [13] is set as the threshold in our study:

(5) $$\bar{c}(t)=\{\begin{array}{cc} \frac{\bar{c}(t-\Delta t)+c(t)}{2} & t\geq \Delta t\\ c(t) & t=0 \end{array}$$

where *c*(*t*) is the output value of the gas sensor at the time *t. c̄*(*t*) and *c̄*(*t* − Δ*t*) are the moving average value, *i.e.*, the threshold, at the time *t* and *t* − Δ*t*, respectively. Thus, the amplitude threshold can be adapted automatically to the accumulation of odor concentration. If the odor accumulates in a certain area, the concentration threshold increases accordingly, and *vice versa*.

**Figure 3.**
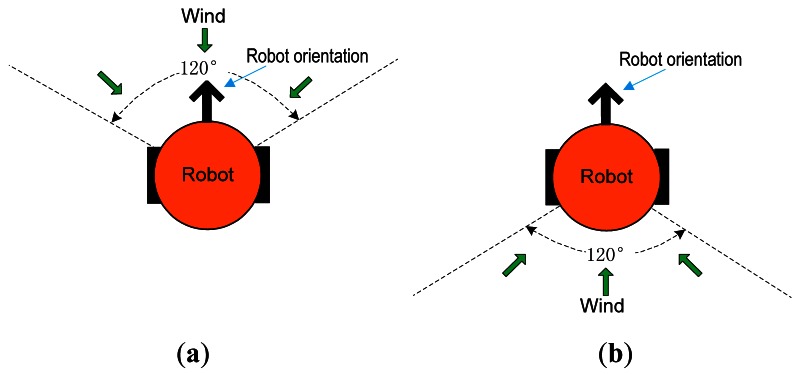
Definition of upwind and downwind directions based on the robot orientation and the sensed wind directions. The solid black arrow stands for the robot orientation, and the green arrows represent the wind directions. (**a**) Upwind; (**b**) Downwind.

As mentioned in [5], an “ATTRACT” communication between robots is used to hold the group together. The detailed rule of “ATTRACT”, however, was not explained. In this paper, the ATTRACT is conducted as follows. If the robot faces the wind direction (see Figure 3(a)), we say that the robot is in the upwind direction. Otherwise, the robot is in the downwind direction (see Figure 3(b)). If the robot is surging upwind, it can attract all the robots downwind or with no plume information. If a robot is attracted by several other robots, it only moves to the nearest one.

## 5. Real-Robot Experiments

Both the proposed AACO+US algorithm and the SS algorithm could work in a distributed mode. To facilitate the experiments process, a centralized way was used in our real-robot experiments. The sensed gas concentrations and airflow information were sent from each robot to a central workstation, and the control commands were sent from the workstation to each robot, both via wireless communication.

### 5.1. Real-Robot Hardware Platform

**Figure 4.**
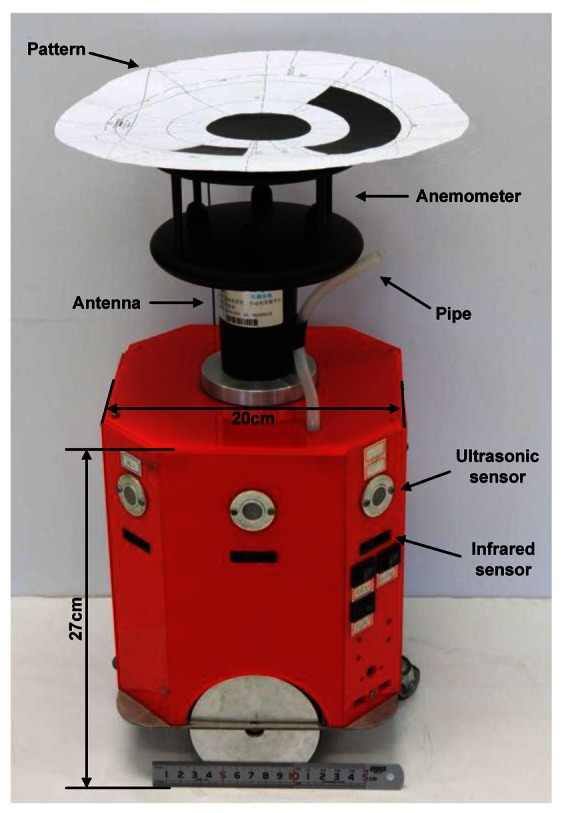
The small mobile robot MrCollie and its onboard sensors.

Four small olfaction robots, named MrCollie, meaning Mobile Robots for Cooperative Odor-source LocaLization in Indoor Environments, were used in the experiments. The robots were designed and assembled by the Institute of Robotics and Autonomous Systems of Tianjin University in 2006. One of the MrCollie robots and its onboard sensors are illustrated in Figure 4. The robot is driven differentially by two wheels, one mounted on the left and the other one on the right. Two castors on the front and back sides are used for balance. The robot is equipped with a two-dimension ultrasonic anemometer (Windsonic, Gill), a gas sensor (TGS2620, Figaro), eight sonar sensors (L Series 40LPT16, Senscomp), eight infrared sensors (GP2D15, Sharp), and a wireless communication module (RPC module, Radiometrix).

The gas sensor is placed in a closed air chamber inside the robot. A small pump and a pipe are connected to the chamber at the back end and front end, respectively. The gas is sucked into the chamber through the pipe. The measured response time and recovery time of the gas sensor in our robot were 0.8 s and 20 s, respectively.

There is a unique location identifier at the top of each anemometer. An overhead charge coupled device (CCD) camera sent the image of each robot's location identifier to the workstation, and the position and orientation of each robot were extracted by the workstation via a simple pattern recognition algorithm. A traffic-rule based method was adopted to avoid robot collision.

### 5.2. Gas Sensor Calibration

The advantage of high sensitivity, long life-span and low cost makes metal oxide semiconductor (MOS) sensors the most widely used gas sensors in mobile robot based OSL. TGS2620, a kind of MOS sensor produced by Figaro Engineering Inc., was used in our real-robot OSL experiments. TGS2620 consists of a silicon semiconductor layer formed on an alumina substrate of a sensing chip together with an integrated heater. In the presence of a detectable gas, the voltage across the heater causes an oxygen exchange between the volatile gas molecules and the metal coating material. Electrons are attracted to the loaded oxygen and result in decreases in sensor conductivity. A simple electrical circuit can convert the change in conductivity to an output signal which corresponds to the gas concentration.

The relationship between the gas concentration and the sensor resistance is expressed as follows:

(6) $$R_{s}=R_{0}{\left(1+\text{a}\times C_{\text{gas}}\right)}^{-b}$$

where *R*s and *R*_0_ represent the sensor resistances in gas and air, respectively; *C_gas_* means the gas concentration; *a* and *b* are constants. A signal processing circuit converts the change in resistance to output voltage *V_out_*:

(7) $$V_{\text{out}}=V_{0}{\left(1+\text{a}\times C_{\text{gas}}\right)}^{b}$$

where *V*_0_ is the output voltage when *C_gas_* = 0.

**Figure 5.**
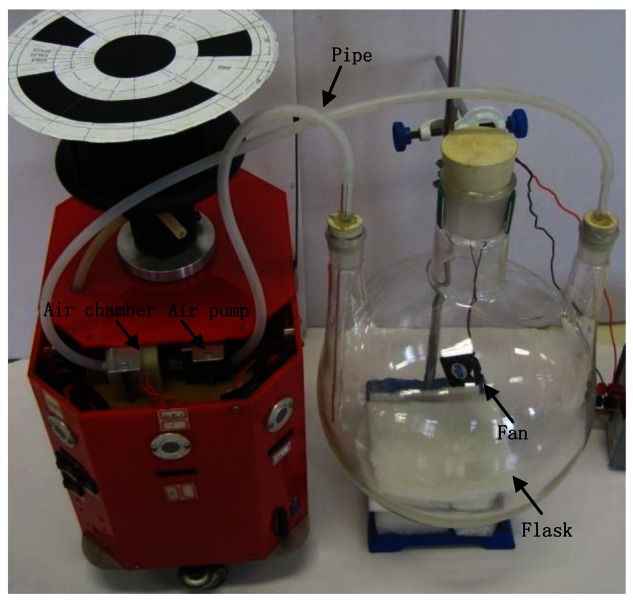
The device for gas sensor calibration. A TGS2620 gas sensor was mounted inside the air chamber.

The calibration process is described as follows: a certain amount of liquid ethanol was injected into a flask, and a fan was employed to speed up the evaporation. The amount of ethanol liquid was calculated according to the desired concentration of the ethanol vapor and the volume of the flask. The vapor was sucked by an air pump into a chamber and contacted with the gas sensor therein. The sensor outputs were recorded after the readings got steady. The calibration device is shown in Figure 5. Through curve fitting, the constants *a* and *b* in Equation (7) could be obtained.

### 5.3. Experiment Arenas

**Figure 6.**
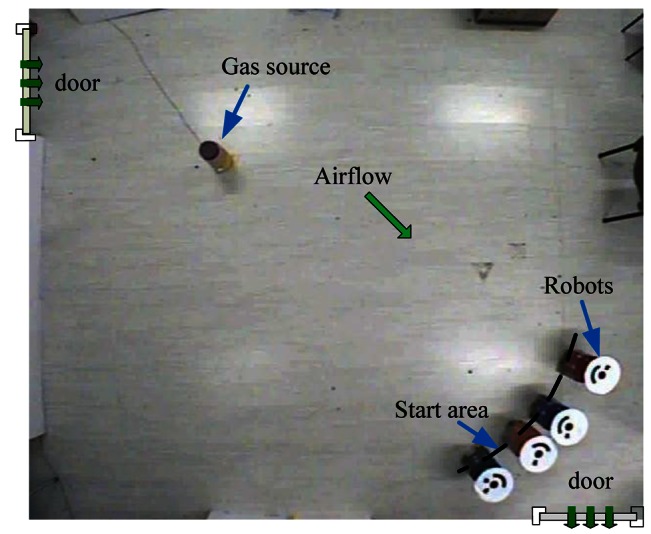
Arena I as seen from the overhead camera.

**Figure 7.**
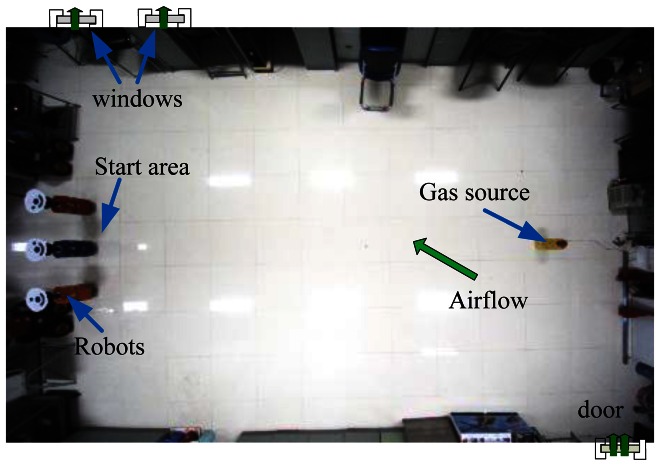
Arena II as seen from the overhead camera.

**Figure 8.**
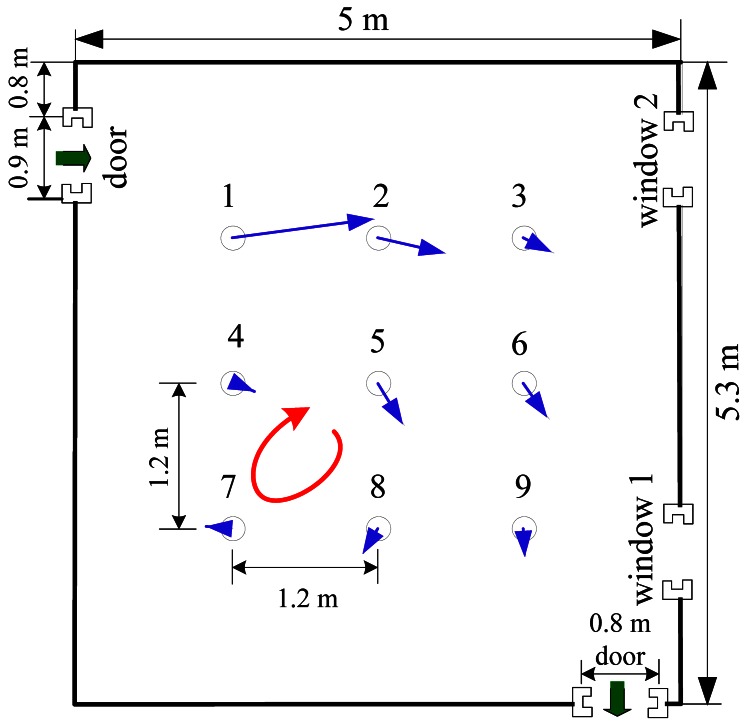
The average natural wind speed and direction of each anemometer over 150 seconds in Arena I. The arrows indicate the directions of the airflow and their lengths indicate the speeds. Notice the large eddy in the lower left corner.

Multi-robot CPT experiments were conducted in two rooms, which are named Arena I and Arena II in our experiments. Figures 6 and 7 show the two arenas seen from overhead cameras. The dimensions of Arena I and Arena II are about 5.3 m × 5.0 m (the detailed dimensions can be seen in Figures 1 and 8) and 9.6 m × 6.5 m, respectively. There are two doors and two windows in Arena I and one door and two windows in Arena II. The height of windows is about 1.2 m off the ground. Along the four sides of two arenas, there are computer desks and chairs, and the central area was left for experiments. In each arena, an overhead CCD video camera was used to localize the robots and record the experiment processes.

A humidifier (see Figures 6 and 7) filled with liquid ethanol was used as the gas source in both arenas. The release rate was 25 mg/s. In Arena I, the gas source was placed in the vicinity of the upper left door, and the robots were initially positioned evenly along an arc. For Arena II, the gas source was placed at the right side, and the robots started from the left side.

### 5.4. Indoor Airflow Field

The natural indoor airflow field in Arena I was created by opening the two doors (with the two windows being closed). Before the CPT experiments, the airflow field was measured using nine two-dimension ultrasonic anemometers (Windsonic, Gill) and analyzed. The layout of wind sensors is given in Figure 8. Each anemometer was 0.4 m off the ground, the same height as the anemometers mounted on the robots. All measured wind speeds and directions were transmitted to a laptop through RS232 cables, with a sampling frequency of 2 Hz.

**Figure 9.**
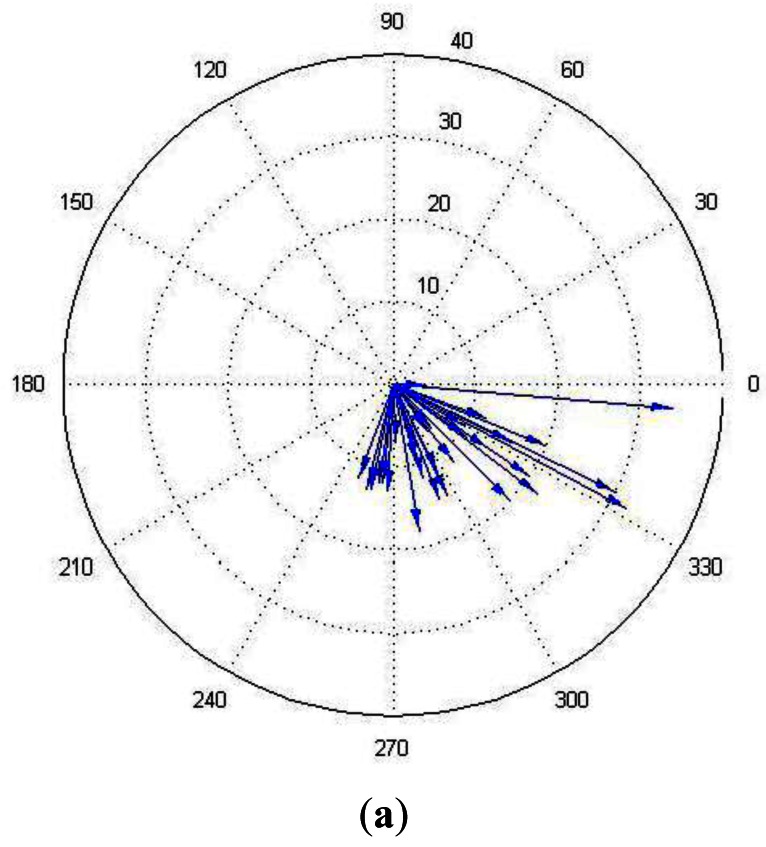

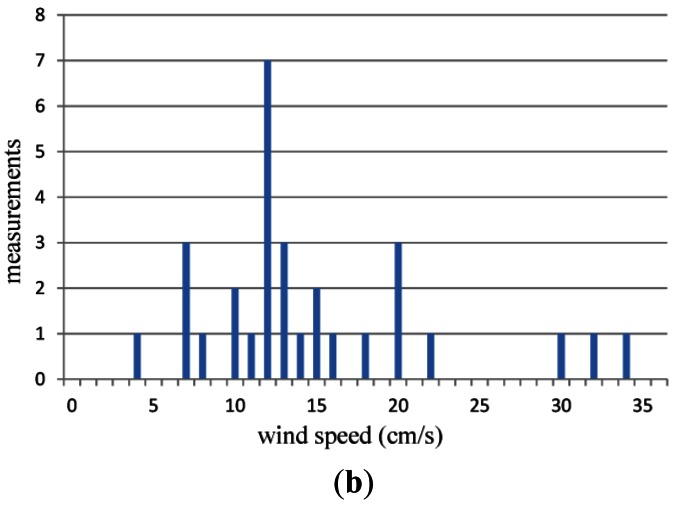
Wind measurements in Arena I by the anemometer 5. The arrows' directions indicate the directions of the airflow and their lengths indicate the speeds. (**a**) Polar plot measurements recorded by the central anemometer; (**b**) Wind speed histogram for the measurements recorded by the central anemometer.

The natural airflow field measured in Arena I is presented in Figures 8 and 9, which show that the natural winds were time and space variant. A large eddy was formed in the lower left corner. Sometimes the wind fluctuated violently and it might blow from the lower right door to the upper left one. Figure 9(a) shows polar plot, which is acquired by the anemometer in the center position (*i.e.*, the anemometer 5). The arrows' directions indicate the directions of the airflow and their lengths indicate the speeds. The wind speed histogram is illustrated in Figure 9(b), which shows the fluctuation of wind speed. For ease of presentation, the measurements displayed in Figure 9 are extracted every 5 s from the total data.

**Figure 10.**
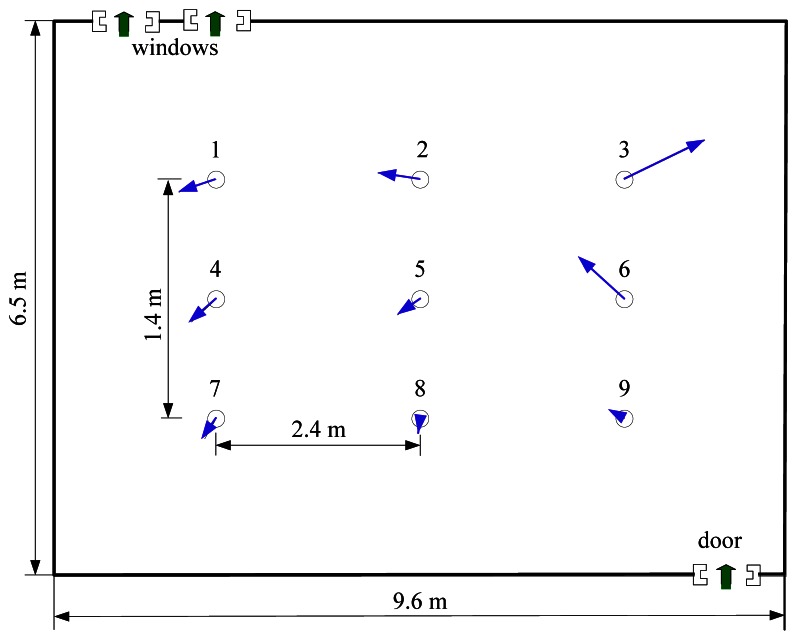
The average natural wind speed and direction of each anemometer over 150 seconds in Arena II. The arrows indicate the directions of the airflow and their lengths indicate the speeds.

**Figure 11.**
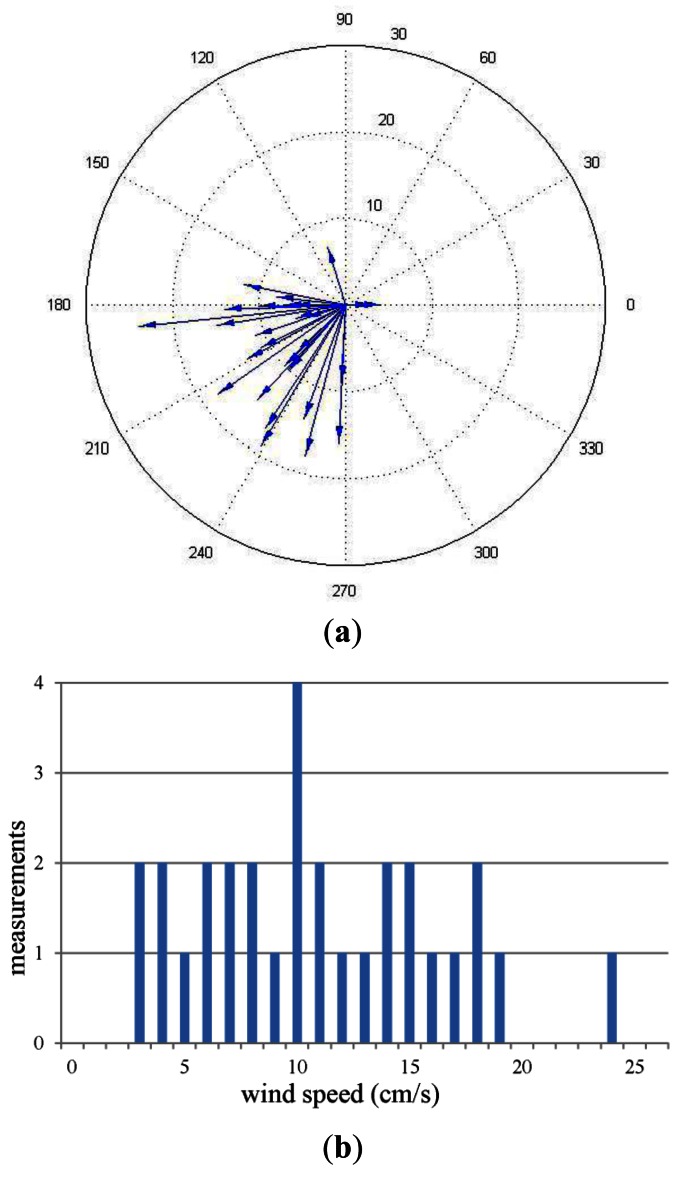
Wind measurements in Arena II recorded by the central anemometer. (**a**) Polar plot measurements recorded by the central anemometer. The arrows' directions indicate the directions of the airflow and their lengths indicate the speeds; (**b**) Wind speed histogram for the measurements recorded by the central anemometer.

Arena II was ventilated by opening the two windows and the door to generate natural airflow. The airflow field was measured in the same way as in Arena I. Figures 10 and 11 illustrate the average wind speed/direction at nine locations and the real-time wind speed/direction measured by the central wind sensor, respectively.

### 5.5. Plume-Tracing Performance Evaluation

Two indexes are proposed for evaluating the CPT performance. One is distance ratio, which is defined as the ratio of the actual average distance traveled by all the robots to the Euler distance between the start point and the gas source. The other index is success rate, which is defined as the percent of the success times out of the total trails. The time of the first robot approaching the source, however, has large randomness. Even multiple robots move randomly, within a period of time it is possible that one of the robots could approach the source. To reduce the chance of random arrival, the success is divided into two cases: one is that any robot approaches a small circular region centered at the gas source within a given time, the other is that all the robots approach the small circular region. The distance ratio reflects the efficiency of the algorithms, while the success rate indicates the robustness.

## 6. Experimental Results and Discussion

In the CPT experiments, the robots moved in a run-stop-run-stop mode (running for 5 s and stopping for 5 s) for both the AACO+US and the SS algorithms. The motion speed of each robot was set to 2.5 cm/s. Both the airflow and gas concentration were sampled five times, once per second, during the 5 s stop.

### 6.1. Experimental Results in Arena I

The group size of robots in Arena I ranged from two to four. For each group size, twenty trials were carried out for the AACO+US strategy and the SS algorithm, so totally one hundred and twenty trials were conducted. The AACO+US and SS algorithms were conducted alternately in order to have the airflow fields for both algorithms keep similar. Each trial was expected to be finished within 900 s (the distance traveled in 900 s by each robot was about three times the distance from the start area to the odor source).

**Figure 12.**
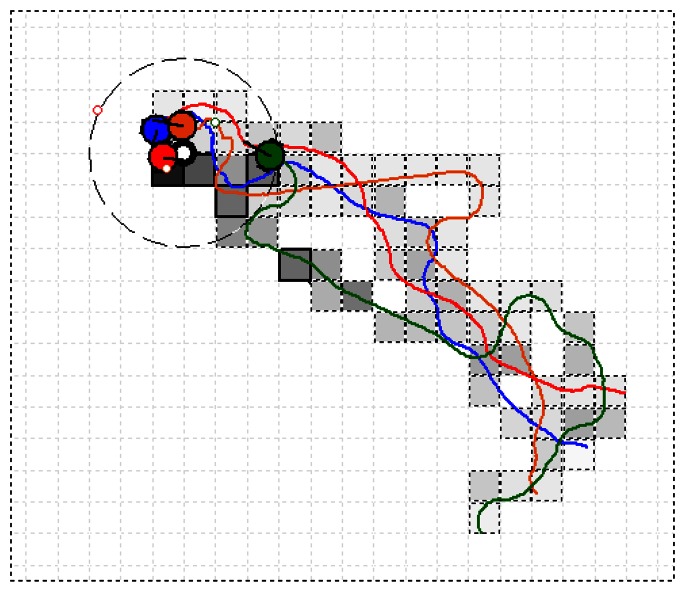
One of recorded CPT processes in the natural airflow field of Arena I using the AACO+US strategy (four robots were used).

Figure 12 shows the user interface and presents one of the CPT processes using the AACO+US strategy in the natural indoor airflow field with a group of four robots. The grids by which the robots passed and in which the measured concentration was higher than a threshold (0 ppm in the experiments) were filled with grey color, with darker color indicating higher concentration. The five maximal-pheromone grids were marked with solid lines. Considering the dimension of MrCollie (see Figure 4), the grid size was set to 20 cm × 20 cm. The large dashed circle centered at the gas source indicated the boundary of the target area. The radius of the dashed circle was set to 60 cm, while the radius of the real gas source was about 10 cm. The robots started from the lower right corner and the trajectories were recorded by the overhead CCD camera. The four robots were expressed as four circles filled in different colors with a short line indicating the attitude angles. In a systematic way, the parameters *C, D, α*, and *β* in (1) were set to 1.50, 500, 5 and 5, respectively.

**Figure 13.**
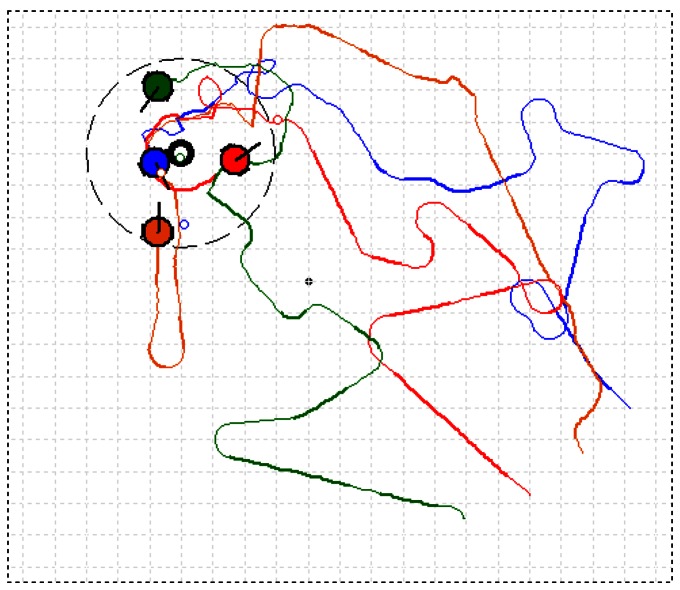
One of recorded CPT processes in the natural airflow field of Arena I using the SS algorithm (four robots were used).

One of the CPT processes using the SS algorithm is shown in Figure 13. The thinner part of the curves suggests no odor packet was encountered in the latest detection, while the bolder part suggests odor packet was detected. On the basis of several optimization trials, the SpiralGap2 and StepSize were set to 35.7 cm and 50.0 cm, respectively.

For ease of presentation, we define a parameter *DR_n_*, which indicates the distance ratio when n robots approach the immediate vicinity of the gas source (*i.e.*, within the 60-cm-radius circle around the source) simultaneously for the first time. *n* is smaller or equal to the group size of robots. We also use ACO-N to indicate the trials carried out by N robots using the AACO+US algorithm, and SS-N using the SS algorithm.

**Figure 14.**
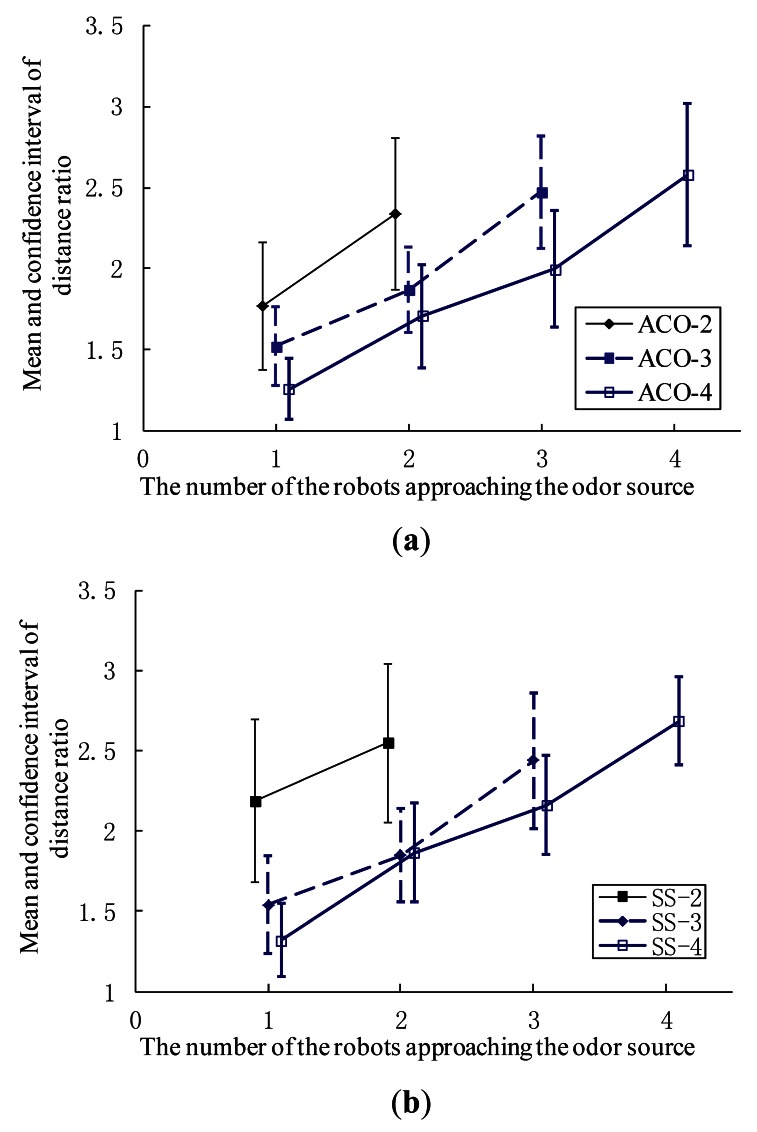
*DR_n_* for real-robot experiments using the AACO+US and the SS algorithms in Arena I. (**a**) AACO+US algorithm; (**b**) SS strategy.

**Figure 15.**
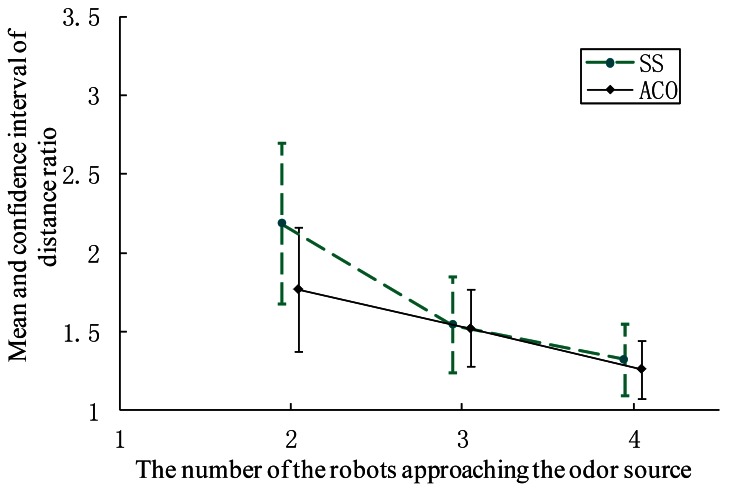
The mean value and confidence interval of distance ratio on condition that the first robot approached the gas source.

**Figure 16.**
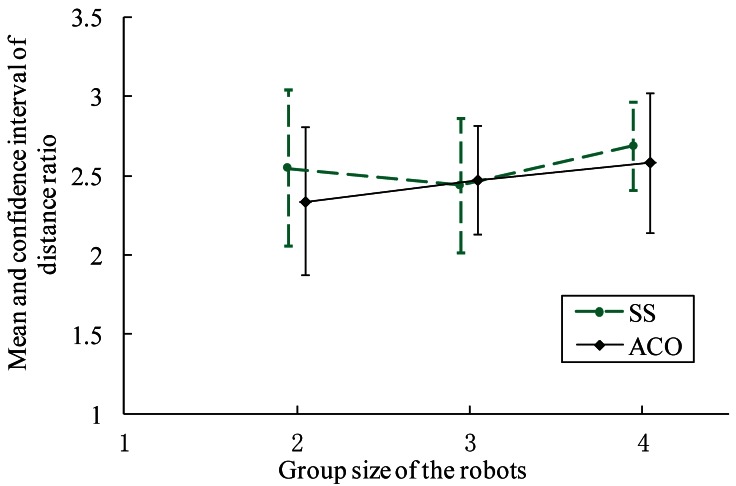
The mean value and confidence interval of distance ratio on condition that the whole robots colony approached the gas source.

Figures 14–16 show comparison of the average distance ratio for real-robot trials using the AACO+US and the SS algorithms in different respects. To make the plots easy to see, the trend lines are horizontally staggered a little. All error bars in the two plots indicate the intervals with 95% confidence level.

Results of *DR_n_* for real-robot experiments in Arena I using the AACO+US algorithm and the SS strategy are illustrated in Figures 14(a,b), respectively. For each n, *DR_n_* decreases with an increase in the group size.

The distance ratio when the first robot approached the gas source is illustrated in Figure 15. For both CPT algorithms, the distance ratio and the corresponding confidence interval decrease with an increase in the group size, that is, multi-robot search improves the efficiency of plume tracing. Furthermore, the AACO+US travels less distance than the SS.

To reduce the randomness, the distance ratio when the whole robot colony approached the gas source was also investigated. As shown in Figure 16, it gets more difficult for all the robots to converge to the target area as the group size increases. The AACO+US algorithm, however, still performs better than the SS.

**Figure 17.**
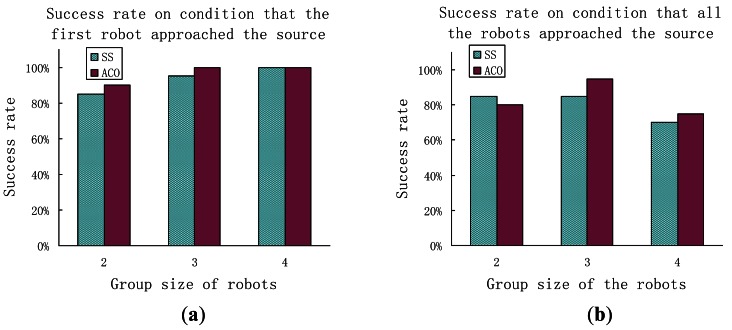
The success rate for the CPT task in Arena I: (**a**) Success rate on condition that the first robot approached the source; (**b**) Success rate on condition that all the robots approached the odor source.

The success rates for different experimental conditions are presented in Figure 17. Both algorithms have high success rates for the CPT task. The success rates increase with an increase in the group size on condition that the first robot approached the odor source but decrease in general on condition that the whole robots colony approached the odor source. The AACO+US shows a little higher success rates than the SS algorithm on the first condition. On the second condition, however, the AACO+US has the highest success rate when the group size is 3, and has lower success rate than the SS as the group size is 2. That might be due to the relatively larger standard deviation of the search time.

### 6.2. Experimental Results in Arena II

The relation between the number of robots and the CPT performance has been demonstrated in Arena I, in this section we only want to show the performances of both CPT algorithms in different indoor environments. To shorten the experimental time, only three robots were employed in the CPT trials in Arena II. Twenty trials were carried out for each of the AACO+US and SS algorithms. The parameters were set the same as those in Arena I except that the StepSize for SS was set as 91 cm. The search time for Arena II was limited to 1,500 s.

**Figure 18.**
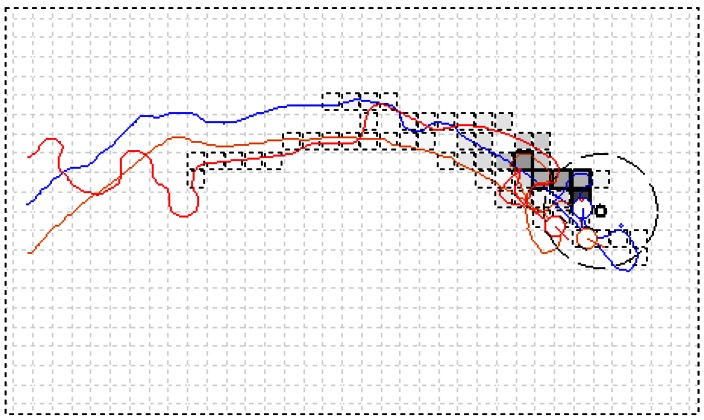
One of recorded CPT processes in the natural airflow field of Arena II using the AACO+US strategy (three robots were used).

Figure 18 shows one of the CPT processes conducted in Arena II by adopting the AACO+US algorithm. The robots started from the left side of the search area, and converged to the gas source finally. By analyzing the trajectory of the blue robot, it can be found that after entering the small circular region of the gas source, the blue robot detected relatively higher concentration and went on surging upwind. Gradually, however, it lost contact with the plume. Then, the ACO part of the AACO+US algorithm helped it to run back to the high-pheromone region.

**Figure 19.**
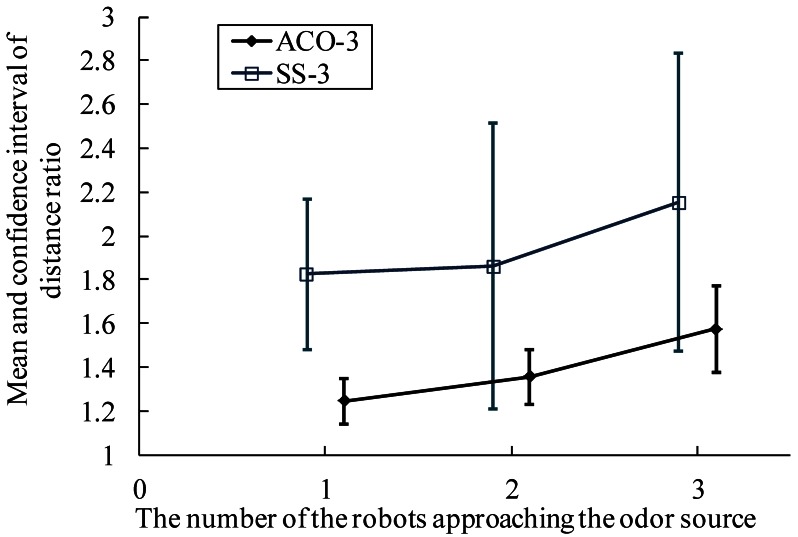
*DR_n_* for real-robot experiments using the AACO+US and the SS algorithms in Arena II.

Results of *DR_n_* for real-robot experiments in Arena II using the AACO+US algorithm and the SS strategy are illustrated in Figure 19, from which it can be seen that the AACO+US has lower distance ratio than the SS. By using AACO+US algorithm, the success rate is 100% on condition that the first robot approached the gas source within the given time (1,500 s); on condition that all the three robots converged to the gas source, the success rate is 70% (14 successes out of 20 trials). For the SS algorithm, the success rates on condition that the first robot approached and that all the three robots approached were 90% and 65%, respectively.

### 6.3. Discussion

The AACO+US algorithm and the SS strategy employ different modes of cooperation among robots. The AACO+US algorithm works by division of labor. The robots are dynamically divided into two subgroups: one subgroup carries out the adapted ACO algorithm, and the other one surges upwind. The robots communicate indirectly via pheromone. In contrast, each robot runs a complete CPT algorithm and can accomplish the task independently in the SS strategy. Only simple “ATTRACT” communication between robots is used to increase efficiency. Based on the analysis and the observation during the trials, it is found that the AACO+US algorithm has better cooperation mechanism than the SS strategy does. That might be one of the reasons why the AACO+US shows higher efficiency than the SS strategy.

Wind is a key factor for the successful tracing of the gas plume in the two algorithms. Because the gas is released from the source and dispersed downwind, moving upwind upon odor-packet detection is an intuitive action for the robot to approach the source. As the airflow fluctuates violently both in amplitude and direction, the CPT performance becomes worse significantly owing to the upwind-search mechanism of the both algorithms. The search trajectories get more meandering.

The SS strategy embodies the spiral and the surge behaviors. The outward spiral pattern behavior, which is designed to find the lost odor packet via local search, cannot guarantee that the robot moves toward the gas source. The surge behavior, which makes the robots search upwind, probably searches straight toward the source. Nevertheless, the surge direction is determined by the instantaneous wind information. Therefore, it might lead the robot opposite to the gas source due to the frequent shifting wind direction. As for the AACO+US algorithm, it divides the robots into two subgroups according to the sampled concentration. The subgroup implementing the adapted ACO algorithm exploits the historical and colonial information to go back to the previous high-pheromone regions, where, however, is usually farther from the gas source. The upwind-surge subgroup serves the similar function as the surge behavior of the SS does, and shares the same weak points. Therefore, both the AACO+US and the SS strategies require relatively steady airflow and perform better in such wind field. Weak airflow combined with rapidly shifting wind directions is a big challenge to both of them.

On the basis of the observations of the experiments, it is found that the frequent obstacle-avoidance behavior reduces the efficiency of the search in the relatively small arena, especially as the group size increases. More robots may further cause worse performance. To speed up the search process, more efficient obstacle avoidance or path planning strategy is preferred. Another way to raise efficiency depends on the inner mechanism of the coordination algorithm, that is, the algorithm itself does not cause many collisions among robots. Reassignment of the goal points at each iteration to minimize the total distance [29] or the maximal time could also work, whereas it has two limitations. Firstly, the gas sensors should read the same value in the same condition. Secondly, this approach depends on the characteristic of the algorithm used. For example, the algorithm should have no special requirement on the path to the goal points. The gas sensors' slow response/recovery and the resulting run-stop-run-stop moving mode at a low velocity add extra search time in practice.

## 7. Simulation Results in Large-Scale Plume Environments

It is quite challenging for multiple robots to trace meandering plumes in outdoor large-scale airflow environments. CPT experiments in outdoor environments using multiple real robots are hard to carry out owing to the realistic difficulties. For example, it is not easy to exactly localize multiple small mobile robots in outdoor environments. In addition, by using the commonly used gas sensors for CPT research, the gas concentration cannot be reliably measured when the robots are located far away from the source. In this research, the large-scale advection–diffusion simulated plume environment created by Farrell *et al.* [18] was used to verify if the proposed AACO+US CPT algorithm could cope with the plume meander issue.

### 7.1. Basic Simulation Assumptions

The size of the robot is negligible compared with the large scale of the search space (100 m × 100 m). It is assumed each robot is equipped with one gas sensor and one wind sensor. The gas sensor has the same response time and recovery time as that in real-robot experiments. The wind sensor measures wind speeds from 0 to 10 m/s and wind directions from 0 to 359°. Zero-mean Gaussian noise is added to the output of the wind sensor, and the variances of the wind speed and direction are set as 0.05 m/s and 1°, respectively. The sampling frequency of the gas concentration and wind sensors is 10 Hz. Each robot knows its current location and moves at a speed of 0.5 m/s. Gas concentration and wind information data recorded by the robots are sent to a workstation via wireless communication. The motion mode of each robot is planned by the workstation.

### 7.2. The Gas Sensor Model

**Figure 20.**
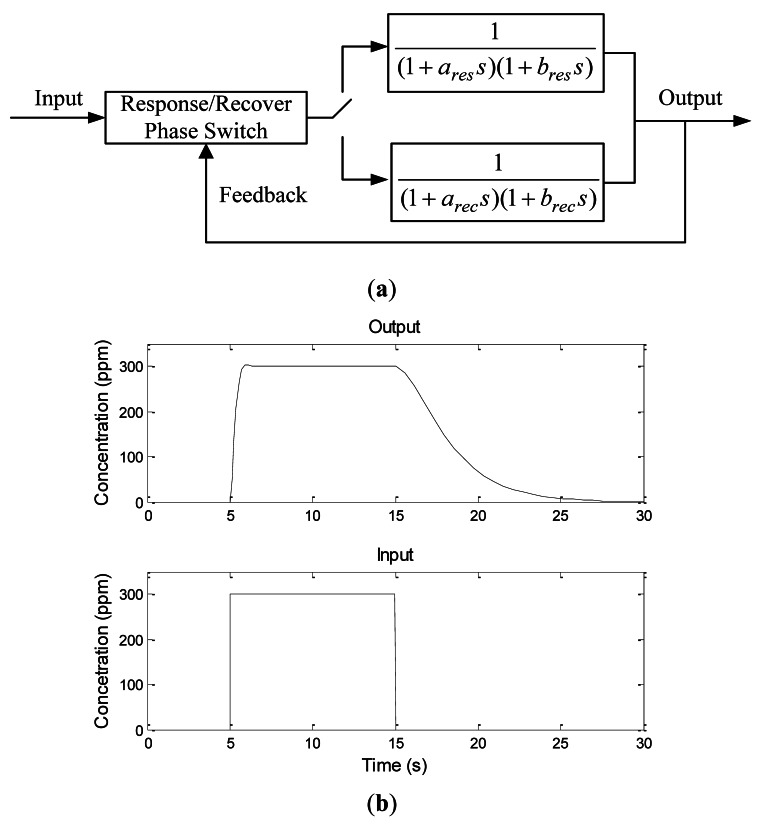
(**a**) The gas sensor model. The second-order transfer functions with different time constants describe the response and recovery phases. The left block is a switch comparing the input and the feedback of the output to decide which phase to choose; (**b**) The response/recovery phase *versus* time.

To simulate the real response and recovery characteristics of metal oxide semiconductor sensors, a second-order sensor model is built here, with the response and recovery phases of the sensors both regarded as second-order inertia links. The two phases have different time constants, and therefore their design parameters are different. The left block in Figure 20(a) represents the switch module for the two phases, and the right two blocks represent inertia links of the two phases. When the output is greater than the input, the recovery phase is chosen; otherwise, the response phase is chosen. Gaussian noise added in the sensor response and recovery phases. In our simulation, the response and recovery time are set to 0.8 s and 20 s, respectively. The response and recovery phases simulated by the sensor model are shown in Figure 20(b). As a step signal is given to the input terminal of the model, the output increases gradually over time and finally keep the same as the input. This is the response phase. When the step signal disappears, the output decreases slowly with the time, *i.e.*, the recovery phase.

### 7.3. Simulation Results

The size of the simulation environment was 100 m × 100 m. Each square grid of the environment was 0.5 m × 0.5 m. The rate of puff release by the source was 5 puffs/s. The search algorithm repeated once per second. The plume-model update period was 0.01 s. The wind speed range was between 0.5 and 2.5 m/s. The gas source was located at (20, 0) and the robots started at (90, −30), where the coordinate unit was meter. The AACO+US CPT algorithm was demonstrated for three different plume environments, which we referred to as slightly wandering, medium-wandering and greatly wandering plumes. The extents of the three plumes in the vertical direction were 20, 60 and 100 m (measured at x = 100 m), respectively.

**Figure 21.**
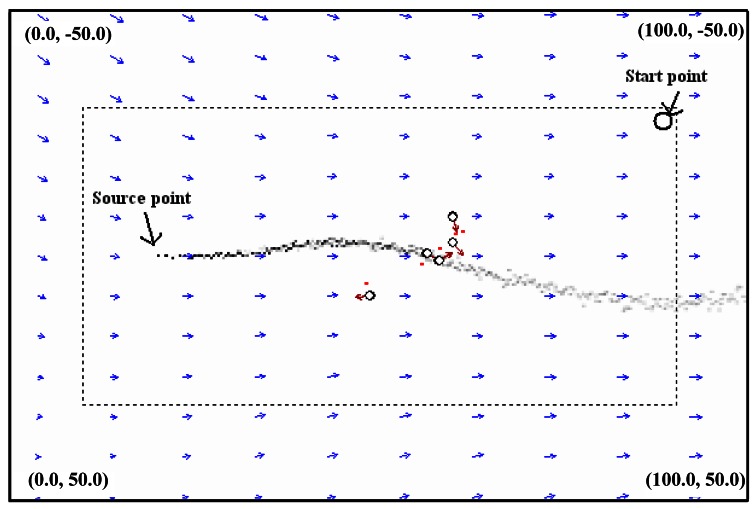
The CPT scene at time *t* = 420 s for the medium-wandering plume environment (five robots are used).

Figure 21 shows the instantaneous CPT scene at time *t* = 420 s for the medium-wandering plume environment using five robots. Each red arrow indicates the direction of the motion of the robot. The blue arrows represent the wind, with the lengths of the arrows denoting wind strengths and the directions indicating wind directions. The five red points represent the maximal-pheromone-concentration grids at the current time.

**Figure 22.**
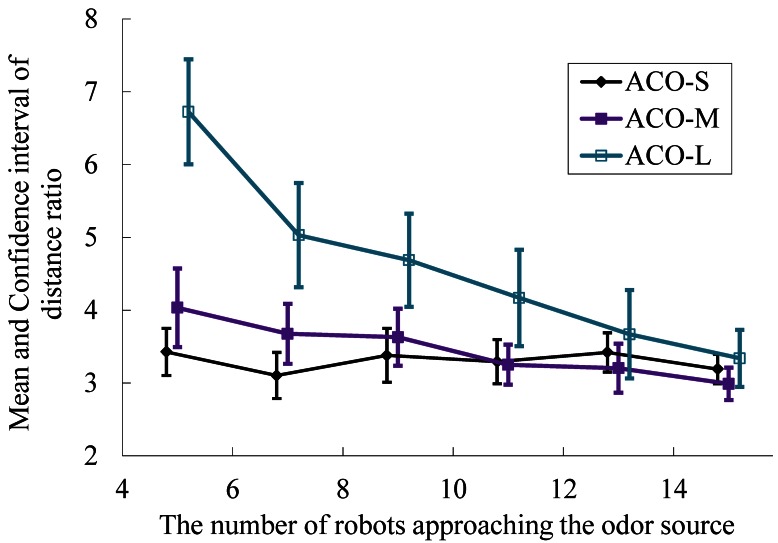
The mean and confidence interval of the distance ratio for different group size in the slightly wandering, medium-wandering and greatly wandering plume environments.

For each group size, twenty five trials were run for each of the slightly wandering, medium-wandering and greatly wandering plume environments. The statistical results are illustrated in Figure 22, in which the abscissa is the number of robots and the vertical ordinate expresses the distance ratio with a 95% confidence level. The symbols ACO-S, ACO-M and ACO-L indicate search results using the AACO+US method for the slightly wandering, medium-wandering and greatly wandering plume environments, respectively. The robots converge near the gas source with a 100% success rate on the condition that at least one robot approaching the odor source.

From the simulation results it can be seen that with an increase in the number of robots, both the distance ratio and confidence interval decrease. When there are relatively few robots, the robots travel longer to find the source in the greatly wandering plume environment. Using more robots, the search times for the three plume environments tend to be the same. Therefore, the AACO+US method has good robustness regarding different plume environments.

## 8. Conclusions

The adapted ACO combined with upwind surge strategy for chemical plume tracing proposed in this paper is investigated and compared with the spiral surge algorithm via multiple real robots experiments in two indoor natural airflow environments. The experimental results demonstrate that the efficiency and robustness of the proposed CPT algorithm are better than those of the SS algorithm. The results of simulations for large-scale advection-diffusion plume environments validate the feasibility of the proposed CPT algorithm in meandering gas plumes. In both the real-robot experiments and computer simulations, the local optimum phenomenon of the traditional ACO did not occur in the proposed AACO+US algorithm.

Both the AACO+US algorithm and the SS strategy require a relatively steady airflow. The frequently changed wind direction makes it more difficult for the robots to trace the gas plume. With an increase in the fluctuation of wind direction, the chemical plume tracing performance becomes worse.

In addition, the experiments also demonstrate that the CPT performance could be improved with an increase in the number of the robots. The proper cooperation among the robots helps increase the efficiency. Meanwhile, however, the performance loss caused by the collision-avoidance action among the robots in the confined search area also becomes significant as the group size increases. Even worse, it might be greater than the performance gain from cooperation, that is, more robots might not promise better performance. Therefore, reducing the internal inference of the robot swarms, e.g., collision avoidance, is a promising way of better performance.

How to utilize the frequently fluctuating wind information is a challenging and significant issue worthy of further study. It is one of the key factors to improve the performance of the AACO+US algorithm. We will also try to extend the AACO+US algorithm for multiple gas source localization in the environments with obstacles in our future work.
